# Pulmonary fibrosis following household exposure to asbestos dust?

**DOI:** 10.1186/s12995-014-0039-0

**Published:** 2014-11-18

**Authors:** Joachim Schneider, Bernd Brückel, Ludger Fink, Hans-Joachim Woitowitz

**Affiliations:** Institute and Outpatient Clinic for Occupational and Social Medicine, University Medical Center Giessen, Aulweg 129/III, D-35385 Giessen, Germany; Institute of Pathology and Cytology, UEGP, Forsthausstr. 1, D-35578 Wetzlar, Germany

**Keywords:** Asbestos, Lung fibrosis, Indoor air pollution, Household contact, Lung dust fibre burden

## Abstract

An 81-year-old woman was dying from histologically confirmed pulmonary fibrosis without having had any asbestos exposure in the workplace. The lung dust fibre analysis showed significantly increased “asbestos bodies” (AB) (2,640 AB per gram of wet lung tissue) and asbestos fibre concentrations (8,600,000 amphibole fibres of all lengths and 540,000 amphibole fibres with a length ≥5 μm per gram of dry lung tissue). Asbestos exposure was revealed to have occurred during household contact after 27 years of washing her husband’s industrial clothing that had been contaminated by asbestos at his workplace in an asbestos textile factory. Household asbestos dust exposure as a risk or co-factor in the aetiology of the fatal pulmonary fibrosis is discussed.

## Introduction

Over 100 years ago, Murray in London [1] identified the first case of pulmonary fibrosis in an asbestos textile worker. Epidemiologically there is no doubt about the causal relationship between occupational asbestos exposure and the risk of mesothelioma, pleural plaques, parenchymal fibrosis of the lung, and lung cancer. Furthermore, asbestos-induced mesotheliomas in housewives have been observed after para-occupational exposure by cleaning asbestos-contaminated work clothing [2], and small, irregular opacities in X-ray examinations as a sign of asbestosis in housewives without occupational exposure have been reported, albeit rarely for review see [3].

## Case report

The 81-year-old housewife cleaned her husband’s asbestos-contaminated work clothing daily for over 27 years by shaking out, brushing out, and washing. The husband was employed as a mechanical engineer in an asbestos textile factory with about 500 employees in the 1960s. His workplace was at a mill, spinnery, and carding facility. Asbestos dust was all over the factory and consequently in his hair and clothes. Their home was located approximately 2 km away from the plant. The wife and her daughter visited the husband at work about once a month. The woman had been previously employed working at the counter of a post office, but after she married, she became a full-time housewife. The woman did not remember any work-related contact with asbestos.

**Figure 1:**
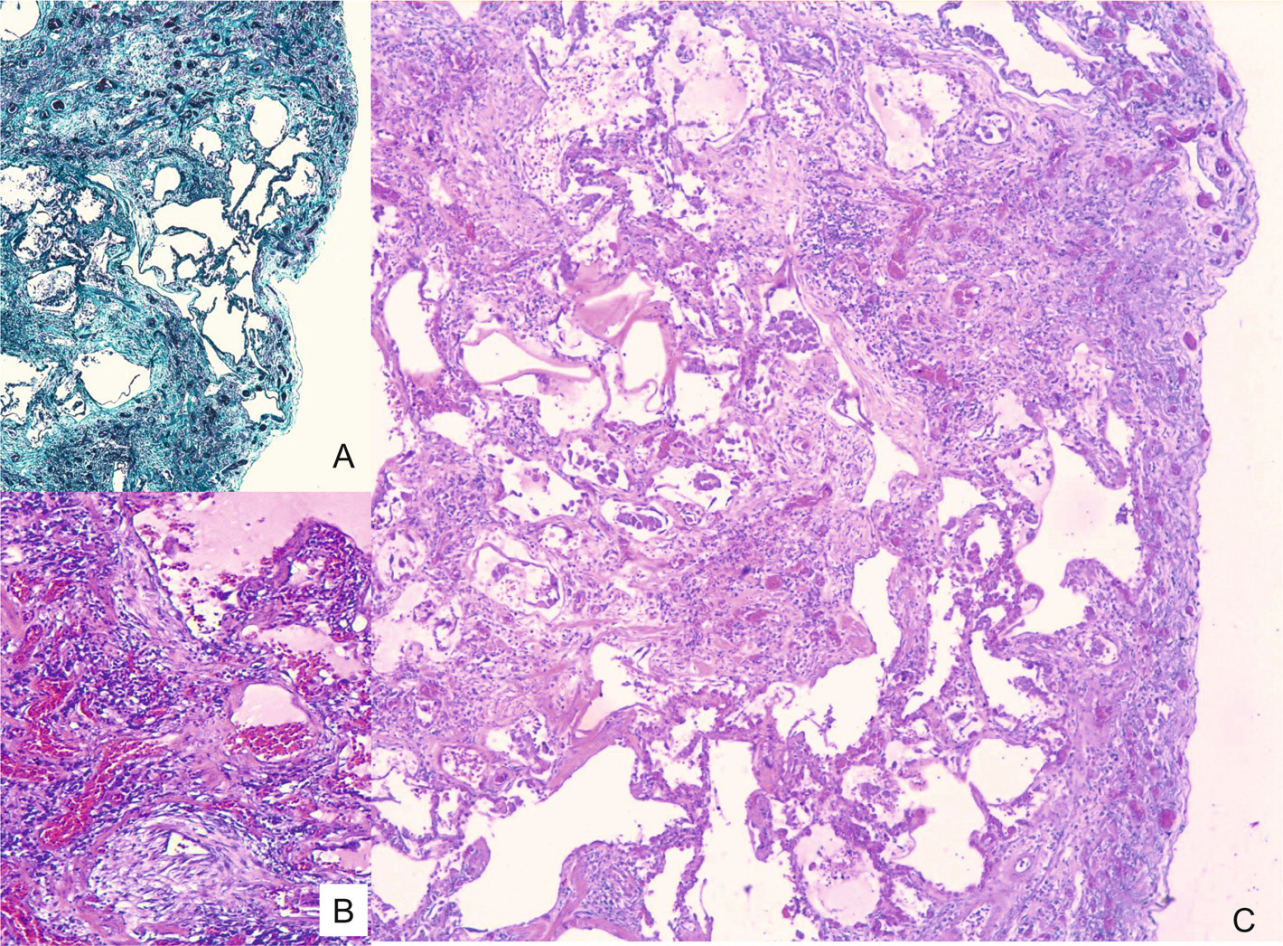
**H&E-stained histological section showing severe interstitial fibrosis with disorganisation of lung structure, temporal heterogeneity with mature scars (insert A, Masson-Goldner staining), immature fibroblastic foci (insert B, 100×), and honeycombing (40×; C).**

**Figure 2:**
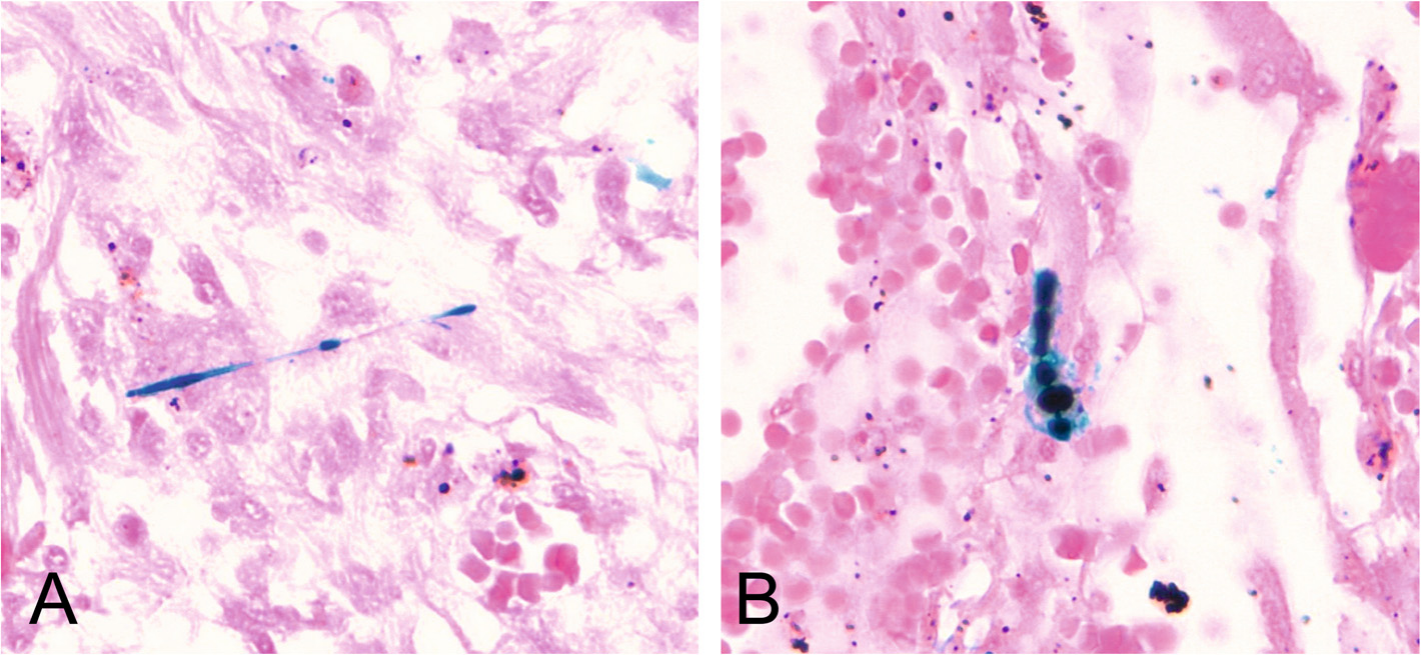
**Scattered asbestos bodies showing a fibrous core coated by iron-containing material within the fibrotic tissue (insert A and B), Prussian blue staining (400×).**

**Figure 3:**
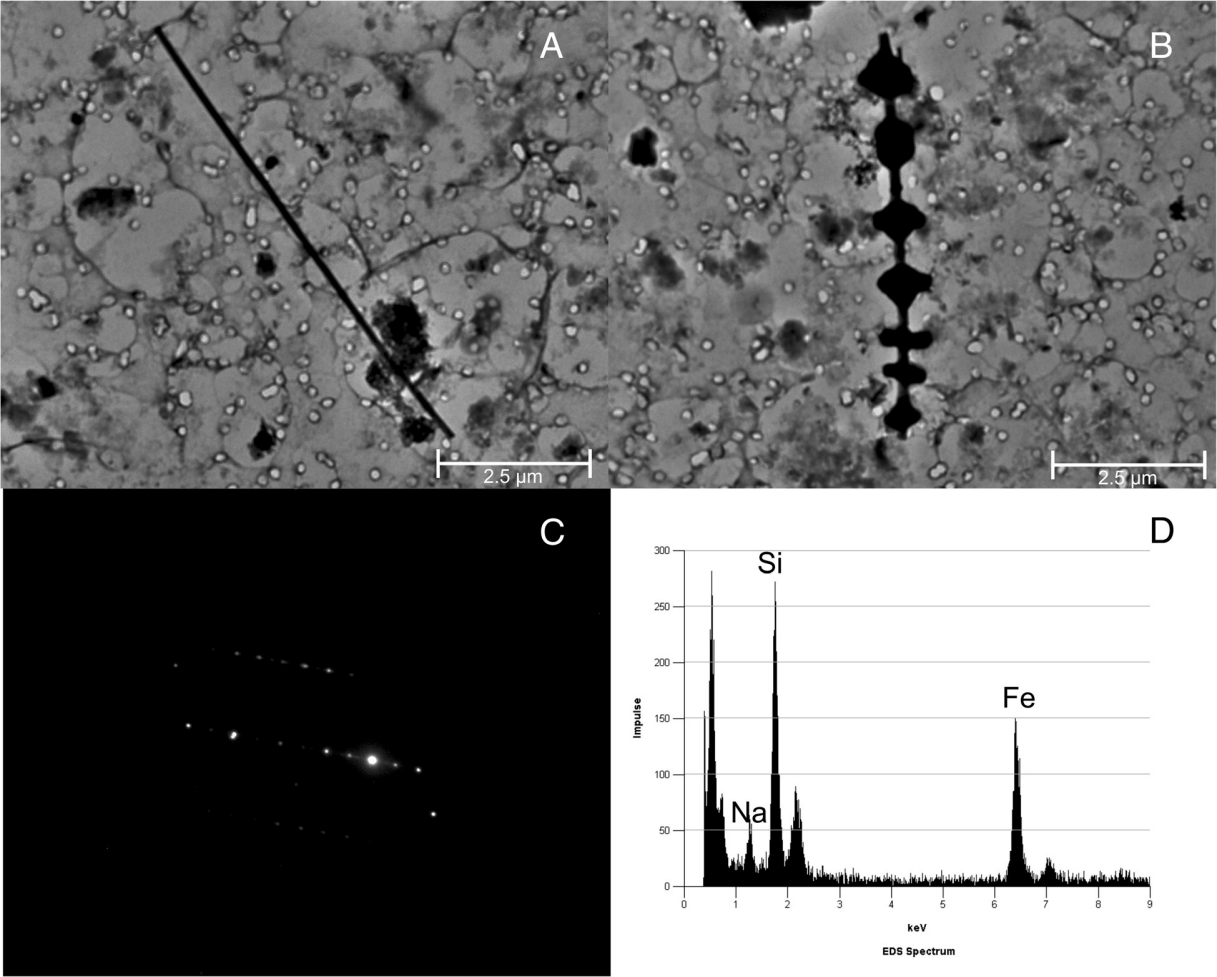
**Lung dust analysis.** Photograph of **A)** a crocidolite fibre, **B)** a ferruginous body (TEM 10,000 x) with **C)** diffraction pattern and **D)** element spectrum.

At the age of 69 she suffered from arterial hypertension, diet-controlled diabetes mellitus, and a myocardial infarction in the septum area. Apart from intermittent feverish bronchitis, there was no further need for medical consultation. After a latency of about 40 years, chest X-rays showed mitral heart configuration and small, irregular opacities s/t in all fields with a profusion of 1/1 according to the ILO classification system. There was diffuse pleural thickening (ILO: 1a) without pleural plaques. Admission to hospital became finally necessary because of progressive respiratory insufficiency. Death was caused by right heart failure with Cor pulmonale and bronchopneumonia. At autopsy, in addition to obliterate coronary sclerosis and chronic bronchitis, extensive pulmonary fibrosis was diagnosed as the underlying disease. Pulmonary tissue showed a partly diffuse and partly small nuclear increase in the connective tissue and a diffuse, reticulated coarsening of the pulmonary texture. The pleura showed diffuse thickening. Histological examination revealed that the lung parenchyma displayed a diffuse interstitial fibrosis with architectural distortion and predominantly subpleural/paraseptal distribution, honeycombing, fibrotic heterogeneity with a few patchy areas of almost intact alveolar septa, and the presence of fibroblast foci mimicking the fibrotic pattern of a usual interstitial pneumonia (UIP) (Figure 1). The severity of fibrosis was grade 3–4 according to a simplified version of the CAP-NIOSH grading scheme [4]. Scattered asbestos bodies (AB) were observed (Figure 2). A total of 2,640 asbestos bodies per gram wet lung tissue were counted after preparing post-mortem lung tissue by a modified NaClO method [5]. By scanning transmission electron microscopy (STEM), including electron diffraction and energy dispersive X-ray spectral analysis (EDX), increased concentrations of amphibole fibres (8.6 × 10^6^ fibres of all lengths and 0.54 × 10^6^ fibres ≥5 μm in length per gram dry tissue) were observed, which were classified as being exclusively crocidolite [5,6] (Figure 3).

## Discussion

Mesothelioma diseases stemming from indoor exposure to asbestos have been described, but the risk due to para-occupational asbestos exposure is still unclear [2]. Whereas the pleura are sensitive to asbestos fibres, it is thought that higher cumulative asbestos doses are needed to cause asbestos-induced pulmonary fibrosis.

Asbestosis is defined as diffuse interstitial fibrosis of the lung as a result of a reliable history of asbestos exposure. The histologically confirmed diagnosis in our case report is consistent with either asbestosis or end-stage idiopathic interstitial fibrosis (cryptogenic fibrosing alveolitis).

In our patient the history indicates a household exposure to asbestos by cleaning asbestos-contaminated work clothing with possible fibre concentrations exceeding 1 fibre/ml [7]. While some authors [8,9] do not see a causal relationship between exposure to asbestos dust outside the workplace and pulmonary fibrosis, others [10] have pointed out that long-term exposure can lead to cumulative fibre retention in the lung that comes close to that of asbestos fibre dust exposure in the workplace.

Pulmonary X-ray abnormalities, which are usually seen only after occupational asbestos exposure, were observed to be more prevalent among family contacts of asbestos factory workers [11] or shipyard workers [12]. With high-resolution computed tomography Candura et al. [13] diagnosed asbestosis in association with calcified pleural plaques in a 68-year-old man and a 72-year-old woman, who lived for several decades in the proximity of a large Italian asbestos-cement plant. The woman also cleaned the work clothes of her brother who worked at the plant.

In the present case, the most likely diagnosis of histologically confirmed pulmonary fibrosis is dependent on lung fibre burden. The distinction between “idiopathic” pulmonary fibrosis and asbestosis is based on conventions concerning the qualitative and quantitative detection of asbestos bodies and asbestos fibres in the lung. The criteria [14] required “the identification of diffuse interstitial fibrosis in well-inflated lung tissue remote from a lung cancer or other mass lesions, plus the presence of either 2 or more asbestos bodies (AB) in the tissue with a section area of 1 cm^2^, or a count of uncoated asbestos fibres that falls into the range recorded for asbestosis by the same laboratory”.

Crocidolite fibre concentrations that we observed in STEM (8.6 × 10^6^ fibres/g_dry_ of all lengths and 540,000 fibres >5 μm/g_dry_ tissue) substantially exceed the limit of 1,000,000 amphibole fibres ≥1 μm and 100,000 amphibole fibres ≥5 μm in length that identify persons with a high probability of exposure to asbestos in the workplace [10]. These values also clearly exceed the upper limit of the normal population (95th percentile) for amphibole fibres of 140,000 fibres/g_dry_ obtained by the same method [6]. The uncoated fibre concentrations as well as the 2,640 AB are within the lower range recorded [15,16] in cases with histologically confirmed asbestosis. In fact, it falls into the range of 33 Wittenoom patients with fibrosis [16].

Mineralogical studies have shown that in patients with asbestosis, the lung burden is 100 to 1,000 times greater than background measurements with respect to the number of AB or amphibole fibres. This appears to contrast with the only slight elevation of asbestos burden in the lung tissue of our patient. The correlation, however, between mineral fibre content of the lung and the severity of fibrosis is somewhat imperfect. Henderson et al. [15] pointed out that a linear dose–response relationship does not exist.

The alternative diagnosis of idiopathic diffuse interstitial fibrosis is questionable because AB are present in tissue sections and the asbestos fibre burden within the lung tissue is higher than the lower 95th percentile of the range for asbestosis reported by Henderson et al. [15]. For these reasons we conclude that this is a case of asbestosis following household asbestos exposure.

## Consent

Appropriate written informed consent was obtained for publication of this case report.

## References

[1] Murray HM: **Report of Departmental Committee on Compensation for Industrial diseases, minutes of evidence.** London: *H.M.S.O* 1907, *Cd.* **3946**: 127–128.

[2] Schneider J, Woitowitz HJ (1996). Tumours linked to para-occupational exposure to airborne asbestos. Indoor Built Environ.

[3] Donovan EP, Donovan BL, McKinley MA, Cowan DM, Paustenbach DJ (2012). Evaluation of take home (para-occupational) exposure to asbestos and disease: a review of the literature. Crit Review Toxicol.

[4] Sporn TA, Roggli VL, Roggli VL, Oury TD, Sporn TA (2004). Asbestosis. Pathology of asbestos-associated diseases.

[5] Rödelsperger K, Woitowitz HJ, Manke J, Brückel B, Giesen T (1985). Die postmortale Lungenstaubfaseranalyse als Beweismittel einer beruflichen Asbestfaserstaubgefährdung. Zbl Arbeitsmed.

[6] Rödelsperger K, Woitowitz HJ, Patrzich R, Brückel B (1990). Asbestfasern und Ferruginous Bodies in der menschlichen Lunge. Teil 1: Asbestfaseranalysen bei weitgehendem Ausschluss einer Asbestfaserstaub-Einwirkung am Arbeitsplatz. Staub Reinh Luft.

[7] Rödelsperger K, Schneider J, Woitowitz HJ (1996). Umwelt- und Innenraumgefährdung durch Asbestfaserstaub außerhalb des Arbeitsplatzes. Gefahrstoffe Reinh Luft.

[8] Weill H (1994). Biological effects: asbestos cement manufacturing. Ann Occup Hyg.

[9] De Vuyst P, Dumortier P, Jacobovitz D, Emri S, Coplu L, Baris Y (1994). Environmental asbestosis complicated by lung cancer. Chest.

[10] Henderson DW, Rantanen J, Barnhart S, Dement JM, Vuyst De P, Hillerdal G, Matti SH, Kivisaari L, Kusaka Y, Lahdensuo A, Langard S, Mowe G, Okubo T, Parker JE, Roggli VL, Rödelsperger K, Rösler J, Tossavainen A, Woitowitz H-J (1997). Consensus report: Asbestos, asbestosis, and cancer: The Helsinki criteria for diagnosis and attribution. Scand J Work Environ Health.

[11] Anderson HA, Lilis R, Daum SM, Selikoff IJ (1979). Asbestosis among household contacts of asbestos factory workers. Ann NY Acad Sci.

[12] Kilburn KH, Lilis R, Anderson HA, Boylen CT, Einstein HE, Johnson S-JS, Warshaw R (1985). Asbestos diseases in family contacts of shipyard workers. Am J Public Health.

[13] Candura SM, Binarelli A, Ragno G, Scafe F (2008). Two cases of asbestosis and one case of rounded atelectasis due to non-occupational asbestos exposure. Monaldi Arch Chest Dis.

[14] Roggli VL (1991). Scanning electron microscopic analysis of mineral fibre content of lung tissue in the evaluation of diffuse pulmonary fibrosis. Scanning Micros.

[15] Henderson DW, Roggli VL, Shilkin KB, Hammar SP, Leigh J, Peters GA, Peters BJ (1995). Is asbestosis an obligate precursor for asbestos-induced lung cancer?. Sourcebook on asbestos diseases.

[16] Dodson RF, O´Sullivan M, Corn CJ, McLarty JW, Hammar SP (1997). Analysis of asbestos fibre burden in lung tissue from mesothelioma patients. Ultrastruct Pathol.

